# w-HAR: An Activity Recognition Dataset and Framework Using Low-Power Wearable Devices

**DOI:** 10.3390/s20185356

**Published:** 2020-09-18

**Authors:** Ganapati Bhat, Nicholas Tran, Holly Shill, Umit Y. Ogras

**Affiliations:** 1 School of Electrical Engineering and Computer Science, Washington State University, Pullman, WA 99164, USA; 2 School of Electrical, Computer, and Energy Engineering, Arizona State University, Tempe, AZ 85281, USA; natran1@asu.edu; 3 Lonnie and Muhammad Ali Movement Disorder Center, Phoenix, AZ 85013, USA; Holly.Shill@DignityHealth.org; 4 Department of Electrical and Computer Engineering, University of Wisconsin-Madison, Madison, WI 53706, USA; uogras@wisc.edu

**Keywords:** human activity recognition, online learning, wearable devices

## Abstract

Human activity recognition (HAR) is growing in popularity due to its wide-ranging applications in patient rehabilitation and movement disorders. HAR approaches typically start with collecting sensor data for the activities under consideration and then develop algorithms using the dataset. As such, the success of algorithms for HAR depends on the availability and quality of datasets. Most of the existing work on HAR uses data from inertial sensors on wearable devices or smartphones to design HAR algorithms. However, inertial sensors exhibit high noise that makes it difficult to segment the data and classify the activities. Furthermore, existing approaches typically do not make their data available publicly, which makes it difficult or impossible to obtain comparisons of HAR approaches. To address these issues, we present wearable HAR (w-HAR) which contains labeled data of seven activities from 22 users. Our dataset’s unique aspect is the integration of data from inertial and wearable stretch sensors, thus providing two modalities of activity information. The wearable stretch sensor data allows us to create variable-length segment data and ensure that each segment contains a single activity. We also provide a HAR framework to use w-HAR to classify the activities. To this end, we first perform a design space exploration to choose a neural network architecture for activity classification. Then, we use two online learning algorithms to adapt the classifier to users whose data are not included at design time. Experiments on the w-HAR dataset show that our framework achieves 95% accuracy while the online learning algorithms improve the accuracy by as much as 40%.

## 1. Introduction

Wearable internet of things (IoT) devices have the potential to change the landscape in health and activity monitoring [1,2]. They are already employed for in-home monitoring of movement disorders to provide doctors better insight into their patients’ daily activities [3]. Wearable devices are also used for new applications, including gait analysis, obesity management, and physical activity promotion [4,5]. These applications enable automatic tracking of the activities of users, such as walking, which can then provide valuable insight to both users and health specialists, since self-recording is inconvenient and unreliable. Therefore, human activity recognition (HAR) using low-power wearable devices can revolutionize health and activity monitoring applications.

Recent advances in low-cost motion sensors and mobile computing have fueled interest in human activity recognition [6,7,8,9,10]. For instance, smartphones equipped with accelerometer and gyroscope sensors enable recognition of activities, such as walking, standing, sitting, and lying down [8,11,12]. The activity information is then used for rehabilitation instruction, fall detection of the elderly, and reminding users to be active [13,14]. The successful design of activity recognition algorithms depends critically on the availability of sensor data that captures the activities of interest. Research studies typically employ wearable inertial sensors or smartphones to collect the data while the users are performing the activities of interest. The data is then used to train and evaluate algorithms for activity recognition. However, the data is rarely made publicly available [15]. As a result, it is difficult to reproduce the results and obtain comparisons with existing approaches. Therefore, there is a critical need for open-source datasets that provide a common platform for HAR research.

**Figure 1:**
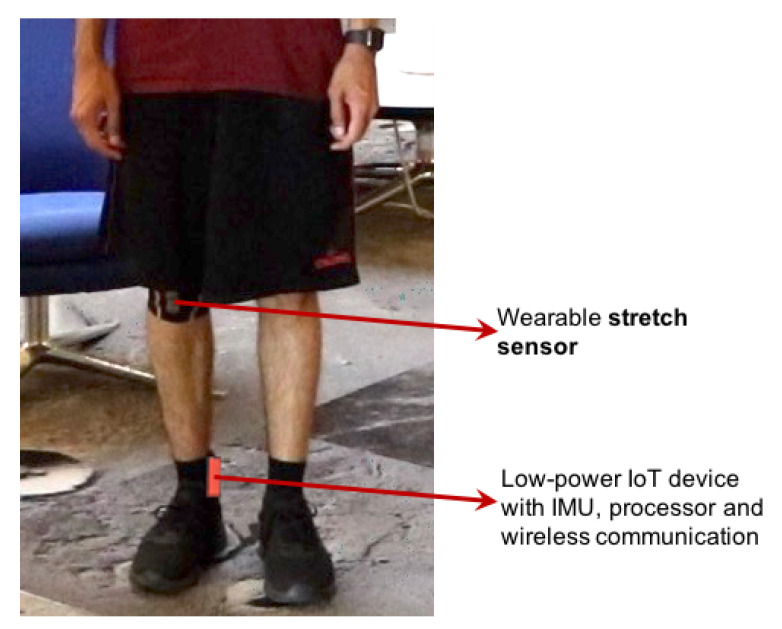
Overview of the wearable setup used for data collection.

This paper first presents wearable HAR (w-HAR), an open-source dataset for HAR. Our dataset is collected using the wearable system shown in Figure 1. It integrates an IMU and textile-based wearable stretch sensors to provide two modalities of motion data. In contrast, other HAR datasets [15,16,17,18] typically use accelerometers and gyroscopes as their primary sensors. However, accelerometers and gyroscopes are notoriously noisy, leading to challenges in data segmentation and classification. The stretch sensor provides low-noise motion data that allows us to generate non-uniform activity segments ranging from one to three seconds. Using the wearable setup, we first perform extensive data collection with 22 user subjects. We record the IMU (accelerometer and gyroscope) and stretch sensor data of each user while they perform activities in the set {jump, lie down, sit, stand, stairs down, stairs up, walk}. Then, we manually label the data such that it can be used to train machine learning algorithms. w-HAR is the first dataset in the literature that includes both IMU and stretch sensor data. The dataset has been publicly released along with this paper to enable further research on activity recognition algorithms.

We provide three versions of the dataset such that users can choose the most appropriate version for their application. The first version includes the raw data obtained from the sensors without any pre-processing. This version is most useful when users want to develop their own segmentation and pre-processing algorithms for HAR, along with feature generation and classifier design. The second version of the dataset uses the segmentation algorithm in [6] to generate variable-length segments. This version allows users to develop their own features and classifiers. Finally, the third version provides the set of features used in our work such that users can focus solely on classifier design.

In addition to the dataset, we also present a comprehensive framework for designing human activity recognition classifiers. Our framework consists of the following steps:

**Design Space Exploration for Offline Classifier:** It is critical to choose a robust and resource-efficient classifier. Resource efficiency is important to ensure that the classifier can execute on wearable devices with power and memory constraints. To this end, we start with commonly used classifiers such as neural networks, random forest, support vector machine (SVM) and k-nearest neighbor (k-NN). Among these, we focus on neural networks, since they can be easily updated online using both reinforcement learning (RL) [19] and supervised learning techniques with low overhead. Once we choose neural networks as our classifier, we perform a design space exploration (DSE) to determine the appropriate structure for the network. The DSE helps us in ensuring that the classifier is robust to multiple sets of users while satisfying the requirement of low resource requirements to run on the wearable device.

**Online Learning:** State-of-the-art approaches for HAR typically train classifiers offline and only perform the activity classification online [7,8]. This approach is not scalable when the device is used by new users with potentially different activity characteristics. Therefore, we also perform online training of the classifiers such that it can adapt to new users who are not involved in the training process. We make use of two approaches to continuously update the weights of the neural network as a function of the feedback available from users. When users can provide the actual activity performed, we incrementally update the policy using supervised learning. Otherwise, we use the policy gradient algorithm [19] when the user can only tell whether the activity is classified correctly. Experiments with our dataset show that these algorithms improve the accuracy of unseen users by as much as 40%.

In summary, the novel contributions of this work are as follows:

- An activity recognition dataset with accelerometer and stretch sensor data from 22 users. It is the first public dataset that includes stretch sensor data for HAR.
- Design space exploration of neural networks to choose the structure of the offline classifier such that it is robust to input from different users.
- An online learning algorithm using incremental supervised learning that provides an order of magnitude faster convergence compared to reinforcement learning. To the best of our knowledge, this is the first time incremental supervised learning has been used to improve the accuracy of classification for new, unseen users.
- An end-to-end framework for HAR development. Most previous approaches provide only algorithms or datasets for HAR. In contrast to these, we provide a customizable framework for HAR, where users have the freedom to insert their algorithms at any step of the framework, including segmentation, feature selection, and classification.
- Experimental validation of the DSE and online learning algorithms on a low-power wearable device using the w-HAR dataset.

We also note that this paper is an extended version of our work in [6]. In comparison to [6], this paper makes the following contributions:

- The conference paper presented only the use of reinforcement learning using the policy gradient algorithm for online adaptation. This paper presents an efficient incremental learning technique that converges an order of magnitude faster than reinforcement learning. We provide a detailed comparison and discuss the advantages and drawbacks of these two approaches in Section 6.
- The conference paper used data from only nine users. This paper includes a labeled dataset from 22 users. Also, the conference paper did not provide the details of the dataset and a public release. This paper provides detailed descriptions of the raw sensor data, segmented data, features, and the experimental protocol for collecting the data.
- This paper includes new activities (stairs up and down) to capture more daily human activities.

The rest of the paper is organized as follows. Section 2 presents the related work. The details of the w-HAR dataset and framework are presented in Section 3. Section 4 describes the design space exploration of HAR classifier, while Section 5 presents the online learning algorithms. Finally, Section 6 and Section 7 present the experimental results and conclusions, respectively.

## 2. Related Research

Human activity recognition has received increased attention in recent years due to its wide-ranging applications in health monitoring, gait analysis, patient rehabilitation, and physical activity promotion [4,20,21,22]. Furthermore, HAR using body-mounted sensors is possible due to advances in sensors and low-power microcontrollers [23]. HAR studies typically start with datasets that are then used to develop the recognition algorithms. With a given dataset, typical steps for activity recognition using sensors include segmentation, feature extraction, and classification.

Several prior studies have presented datasets for HAR [15,16,17,18]. Most of the datasets presented earlier focus on acquiring data from smartphones and performing HAR on them. For instance, Micucci et al. [15] present a dataset for HAR using accelerometers on smartphones that include data from thirty subjects and nine activities of daily living. The authors also present a review of other publicly available datasets collected using smartphones. Wearable sensors have also been used in HAR datasets since multiple devices can be easily mounted on different parts of the body. For instance, the Opportunity dataset presented in [17] uses multiple inertial measurement units and accelerometers to collect data from four users. Similarly, Zhang et al. [18] use a single motion sensing unit to obtain data from 14 users. While these datasets are useful for HAR, they primarily contain data from accelerometers, which is known to be noisy. As a result, studies using these datasets resort to fixed-length windows, instead of creating a window for each activity. In contrast, the dataset presented in this paper includes data from a textile-based stretch sensor and IMU, which allows us to create variable-length segments tailored to each activity.

HAR approaches in the literature typically use fixed-length windows to identify user activities [11,20,24]. For instance, studies in [11,20] use 10 s windows for activity recognition. A longer window length increases the accuracy since it provides more information and features about the underlying activity [22]. At the same time, longer windows make it harder to capture transitions between activities, such as stand to sit. Furthermore, long windows can have data from multiple activities, which leads to inaccurate activity classification [22]. To address this problem, Chen et al. [25] develop a step detection algorithm to segment activities from accelerometer data. However, due to the high noise in the accelerometer data, the algorithm uses a one-second sliding window filter (with a 50% overlap) to mitigate the noise. The filtering mechanism’s increased memory requirement makes the approach in [25] impractical for devices with small memory capacities. Due to the limitations of using accelerometer data for segmentation, Chen et al. [25] also highlight the need for better segmentation algorithms to improve HAR accuracy. Therefore, in our dataset, we use a segmentation algorithm that produces variable-length segments as a function of the user activity [6]. Using these windows, we generate fast Fourier transform and discrete wavelet transform of the data for use in the activity classifier. We include these feature sets as part of our dataset release.

Several studies in the literature proposed classification algorithms for HAR [20,26,27], as summarized in Table 1. This table lists the device used for processing, sensors, classifiers, and accuracy of the HAR approaches. It also lists the power consumption of the approach, when available. Arif et al. [20] use an accelerometer on a smartphone to classify six activities. The authors achieve 95% accuracy using a KNN classifier. Similarly, Ignatov et al. [28] employ CNNs to recognize activities from smartphone accelerometer data. The CNN achieves 90–97% accuracy on three publicly available datasets. The work in [29] uses a wearable device consisting of heart rate, respiration rate, and accelerometer sensors to record the data when the user is moving. Then, the data is transmitted to a smartphone for classification. Using a decision tree classifier, the approach obtains 96% accuracy. Approaches in [26,30,31] use wearables for both sensing and classification. For instance, Attal et al. [26] use three accelerometers and a wearable node to recognize 12 activities. They use three classifiers with accuracies ranging from 95% to 99%. However, this approach has a power consumption of 2.7 W, which is not sustainable for light-weight wearable devices with a small battery size. Samie et al. [30] and Khalifa et al. [31] reduce the HAR power consumption on wearable devices. In particular, Khalifa et al. use a piezoelectric harvester as the sensor, thus allowing them to harvest energy and identify motion simultaneously. As a result, the power consumption reduces to 3.2 mW, albeit at a reduced accuracy of 85%. However, none of the previous approaches support online updates of the classifier for new users. Instead, they only use offline training with the user data available at design time. Offline training alone is not sufficient as it can lead to a lower accuracy when used on unseen users. To overcome this limitation, we present an online learning framework for HAR in this paper. We first train a neural network offline to generate an initial implementation of the HAR system. Then, we use reinforcement learning or incremental supervised learning at runtime to improve the accuracy of the system for new users. Our system has a power consumption of 12.5 mW, which is slightly larger than approaches without any online learning.

Non-contact methods for HAR have also been studied recently [32,33,34,35,36,37]. These approaches use ambient Wi-Fi signals or radars to track user activities. The techniques based on Wi-Fi signals use the channel state information from Wi-Fi signals to infer human activities [34,35,37]. In particular, Taylor et al. [37] use radio signals from a USRP radio system to identify standing up and sitting down. The system uses changes in channel state information from the radio signals to identify the activities. Specifically, it looks at how the channel state information changes due to stand up and sit down motions. The channel state information is then used in machine learning algorithms such as random forest, KNN, SVM, and neural networks. The results from these classifiers are used by an ensemble classifier that identifies the activity using majority voting. Similarly, radar-based approaches emit modulated signals in an indoor environment and analyze the received signals to identify human activities. These non-contact methods are complementary to our approach of using wearable devices. In our vision, both wearable and non-contact methods can be used inside a home setting. However, wearable devices are more suitable for activity monitoring outside the home environment due to privacy issues of user tracking with public Wi-Fi signals. In summary, we envision that our algorithms will enable personalized HAR devices that adapt continuously to the unique activity pattern of their users.

## 3. Human Activity Recognition Dataset

The availability of datasets is crucial for human activity recognition research. Therefore, we open-source our dataset to enable further research in this area. The dataset in this paper is the first to integrate readings from a wearable stretch sensor and an inertial motion unit (IMU). The stretch sensor allows us to create variable-length segments. The variable-length segments make it easier for the classifiers to recognize activities, as we show in the experiments. In this section, we describe the details of the data collection setup, protocol, user demographics, and the labeling process.

### 3.1. Wearable System Setup

We use a combination of Invensense-9250 IMU and stretch sensors to collect the data, as shown in Figure 1. The IMU is integrated into the TI-CC2650 Sensortag device, and the stretch sensor is another discrete module. We mount the IMU on the right ankle of the user since this captures the swing of the user’s leg [12]. The stretch sensor is sewed to a knee sleeve, as shown in Figure 1. During the experiment, the user wears the sleeve on the knee to capture the knee movements while performing the activities. Both the sensor devices are equipped with the Bluetooth low energy (BLE) protocol for communication. Using the BLE protocol, the sensors transmit the data to a smartphone which stores the data to a file. In our future work, we plan to integrate the IMU and the stretch sensor into a single device such that a single stream of data can be transmitted. To synchronize the data from the sensors, we record the wall clock time for each data sample from the sensors. Then, using the offset between the sensors, we align the sensor readings using the approach in [38].

**Wearable System Sensor Parameters:** We sample the IMU at 250 Hz and the stretch sensor at 25 Hz. These sampling frequencies are sufficient to capture the frequency of human movements, which are in the order of a few Hz. We use a significantly higher frequency for the accelerometer since it typically exhibits higher noise. Therefore, the higher sampling frequency allows us to smooth and sub-sample the data using a moving average filter while preserving the data signatures.

### 3.2. User Studies

We obtain motion data from twenty-two users with the wearable device setup. The users are recruited using the snowball sampling technique. Each user signed a consent form as approved by the institutional review board at Arizona State University. We experiment with a total of twenty-two users (consisting of fourteen males and eight females), with ages 20–45 years and heights 150–180 cm. The set of activities performed by the users is summarized in Table 2. Each user performs a series of experiments shown in Table 3. In addition to this protocol, we also perform experiments where the users are free to perform any activities they choose. Next, we perform the labeling of the dataset, as described below.

**Data Labeling:** After collecting the data from the users, we use the segmentation algorithm to divide the data into variable-length windows. Then, the generated windows are analyzed by four human experts to assign the labels. To achieve accurate labeling, we mark the time stamps of each activity during the data collection. For example, we mark the time the subject starts and stops jumping. Furthermore, the labels assigned by one expert are verified by others to ensure that wrong labels are not assigned. The labels assigned to each activity window are also assigned to each sample in the raw data before segmentation such that we know the user’s activity in each sampling period. Moreover, we revisit the assigned labels during the testing phase of the HAR classifier to ensure that the errors made by the classifier are not due to mislabeling.

### 3.3. Dataset Description

After labeling the data, we generate three versions of the dataset for public release as follows.

**Raw Data:** This version of the dataset contains the raw data obtained from the stretch and IMU (accelerometer + gyroscope) sensors without any pre-processing. We synchronize the stretch and IMU data such that the time indices for both sensors are aligned. Consequently, users do not have to run any synchronization algorithms on the data. The raw data version of w-HAR is ideal for researchers who want to design their algorithms for all steps of HAR from segmentation to classification.

**Segmented Data:** The segmented dataset uses the segmentation algorithm proposed in [6] and summarized in Section 3.4.1 to generate variable length activity segments. This version is suitable for users who want to focus on feature generation and classification algorithms. The breakdown of the total segments of each activity is summarized in Table 4.

**Feature Data:** We also release the features used in [6] as part of w-HAR. The features included in this version are summarized in Section 3.4.2. The feature data version allows users to focus on developing classifiers for HAR and obtain reproducible comparisons among different algorithms.

In summary, w-HAR includes a total of 4740 segments with a total duration of about 3 h. The dataset is available for download at our GitHub page: https://github.com/gmbhat/human-activity-recognition.

### 3.4. Flow for Using the w-HAR Dataset

**Figure 2:**
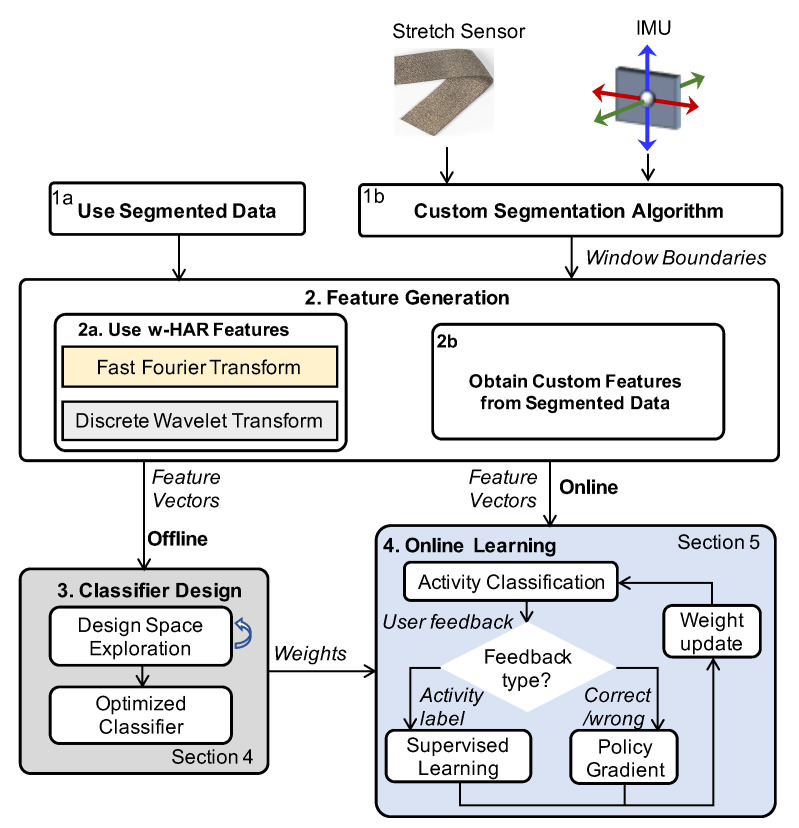
Flow-chart for using the w-HAR data set to test new algorithms or reproduce the results presented in this paper.

This section describes the flow for incorporating the w-HAR dataset for developing new algorithms or reproducing the results reported in this paper, as shown in Figure 2. The first step after obtaining the dataset is to segment the raw data into windows. To this end, users can either use the windows provided along with the dataset or develop their segmentation algorithm, as shown using paths 1a and 1b, respectively. The next step is to generate features for each window generated by the segmentation algorithm. Here, the users are free to generate their features or use the baseline feature set provided with the dataset. The feature data and the labels are then used to design a classifier for activity classification. It involves a design space exploration for determining the optimal classifier, as shown in Figure 2. We also provide a baseline neural network classifier such that it is easy to obtain reproducible comparisons with new approaches. Finally, the last step of the design flow is to use the classifier designed offline to identify activities at runtime. In this step, user feedback is used to update the weights of the classifier to improve the accuracy of the classifier. As shown in Figure 2, our implementation of the framework uses either reinforcement learning or incremental supervised learning depending on the level of user feedback available. When the user provides the actual label of the activity performed, we use the supervised learning approach to update the classifier weights, as shown in Figure 2. However, if the user can only indicate whether an activity is correctly classified, i.e., correct or wrong, then we use the policy gradient approach. We envision that further research on HAR using our dataset will enable new online learning algorithms for personalized activity recognition and healthcare. Next, we describe the segmentation algorithm used to generate the segmented data in w-HAR. We also go over the feature set that is released as part of w-HAR.

#### 3.4.1. Segmentation Algorithm

The length of the activity window must be carefully chosen to capture the activities while minimizing the power consumption of the device. For example, fast activities, such as jump, require shorter windows while static activities, such as sitting, can save power by using longer windows. Fixed-length windows are not suitable for this purpose as they may contain fragments of multiple activities, thus adding to classification complexity. Therefore, we use the activity-based segmentation in [6] to generate the segments in w-HAR. We refer the readers to [6] for details of the algorithm and provide a summary here.

**Figure 3:**
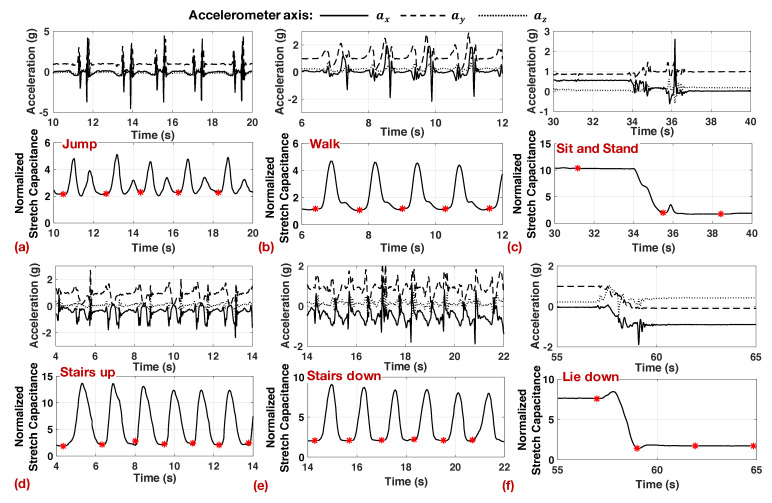
Demonstration of the segmentation algorithm for (**a**) jump, (**b**) walk, (**c**) sit and stand, (**d**) stairs up, (**e**) stairs down, and (**f**) lie down activities in the w-HAR dataset.

Figure 3 shows the sensor data for all seven activities. In each sub-figure, the top half shows the accelerometer data, while the bottom half shows the stretch sensor data. We see that the accelerometer data exhibits significantly more variations when compared to the stretch sensor for activities that involve the movement of the legs (jump, walk, stairs up/down). In contrast, the stretch sensor data exhibits periodic repetitions with each repetition of these activities. Even for static activities (sit, stand, and lie down), the accelerometer exhibits high variation when there is a slight movement of the legs. For the same activities, the stretch sensor follows a smooth pattern, as shown in Figure 3. The variations in the accelerometer data lead to false positives when it is used for segmentation [25]. Therefore, the segmentation algorithm uses the stretch sensor data to obtain segments as a function of the activity.

Using the insights obtained from Figure 3, the segmentation algorithm in [6] divides the streaming data into distinct segments by detecting the deviation of the stretch sensor from its neutral value at rest. For instance, each repetition of jump, walk, stairs up/down in Figure 3a,b,d,e starts with an increase from a local minimum in the stretch sensor and ends with another local minimum. Even for activities with longer static periods (sit, stand, and lie down), the beginning and end of the activity are marked by rise from a local minimum or drop down to a minimum, as seen in Figure 3c,f. Therefore, the algorithm uses this observation to continuously monitor the derivative of the stretch sensor to mark the activity boundaries. The pseudo-code for the algorithm is presented in the Appendix A for interested readers.

The segments obtained from the algorithm for all the activities are shown in Figure 3 using red asterisks. The algorithm clearly marks each step in jump, walk, and stairs up/down activities. For sit, stand, and lie down activities, the algorithm uses a 3 s window whenever the sensor data is static. At the same time, whenever there are transitions, such as from sit to stand in Figure 3c, the segmentation algorithm detects this and marks a new segment for the transition.

#### 3.4.2. Feature Set in w-HAR

The next step after segmenting the streaming data is to generate features that are used by a classifier. As shown in Figure 2, users of w-HAR can use the default feature set provided with the dataset or develop their own feature set. Here, we provide a summary of the features provided in the dataset, while the detailed motivation for choosing these is presented in [6]. We generate features from the accelerometer and stretch sensor data present in w-HAR. We do not use the gyroscope data since it does not provide accuracy improvements, even though it has up to 10 mW overhead in power consumption at runtime. At the same time, the w-HAR release includes gyroscope data such that other researchers can use it when developing their classifiers for HAR. The features described in this section can be used by researchers directly to train a classifier, or the underlying feature generation algorithms can be used along with a custom segmentation algorithm.

**Stretch sensor features:** The stretch sensor exhibits repetitive patterns for walking, jump, stairs up, and stairs down activities, as shown in Figure 3. In contrast, it shows a steady value for sit, stand, and lie down activities. The segmentation algorithm divides these activities into windows that range from 1 s to 3 s in length. As a result of this, the number of samples in each window varies with the length of the window. At the same time, feature generation blocks typically expect a fixed number of input samples for processing. Therefore, as the first step in feature generation, we standardize the data to ensure that each window contains a fixed number of samples. We choose to maintain 32 samples for the stretch sensor data after sub-sampling. This translates to a sampling rate of 10 Hz for windows with 3 s duration and 32 Hz for windows with 1 s duration. These sampling frequencies are sufficient since human activities are typically in the order of a few Hz. When a segment has more than 32 samples due to either longer duration or higher sampling frequency, we standardize the number of samples to 32 using the sub-sample and smoothen equation below:

(1) $$s_{s}\left[k\right]=\frac{1}{2S_{R}}\sum_{i=-S_{R}}^{S_{R}}s\left(tS_{R}+i\right),\;0\leq k<32$$

where $S_{R}=\left\lfloor N/32\right\rfloor$ is the subsampling rate, and $s_{s}\left[k\right]$ is the value of the data after applying the sub-sample and smoothen equation. If the number of samples in a segment is lower than 32, we pad zeroes to the segment to obtain 32 samples.

Next, we evaluate the FFT coefficients of the current and previous windows to capture the repetitive patterns present in the data. More specifically, we use the leading 16 FFT coefficients of the 64-point FFT of the stretch sensor data. The leading coefficients allow us to capture the frequency range that has most of the energy in the data, i.e., [0–8] Hz. In addition to FFT coefficients, we include the minimum and maximum value of the stretch sensor in each window as it provides useful insight into the underlying activity. Overall, we include a total of 18 features from the stretch sensor data.

**Accelerometer features:** The accelerometer data exhibits higher frequency variations when compared to the stretch sensor, as seen in Figure 3. Therefore, we maintain $2^{6}=64$ points of accelerometer data in each activity segment. We use Equation (1) to sub-sample and smoothen the data when there are more than 64 samples in the segment. The three-axis accelerometer in our experimental setup provides the acceleration $a_{x}$, $a_{y}$, and $a_{z}$ along x-, y-, and z-axes, respectively. In addition, the body acceleration $b_{acc}=\sqrt{a_{x}^{2}+a_{y}^{2}+a_{z}^{2}}-g$ after removing the gravitational acceleration *g* is also computed since it provides useful information about the user activity. After standardizing the accelerometer data, we calculate the first level approximation coefficients $A_{1}$ using the Haar discrete wavelet transform, which corresponds to the 0–32 Hz frequency range. With 64 samples in each window, this amounts to 32 approximation coefficients. In the w-HAR dataset, we use the DWT coefficients for $a_{x}$, $a_{z}$, and $b_{acc}$. We do not take the DWT of the $a_{y}$ axis since we do not expect any activity in the lateral direction. Furthermore, the effect of any lateral movement is already captured by the body acceleration. In addition to the DWT coefficient, we also compute the variance of $a_{x}$, $a_{y}$, $a_{z}$, and $b_{acc}$ and use them as features. Overall, the dataset includes a total of 101 features for the accelerometer data.

**General features:** The time duration of the segment also carries important information about the activity. In particular, it provides information on the time scale of the activity, which is otherwise not available after normalizing the number of data points in each segment. Therefore, we add it to our feature set to obtain a total of 120 features.

After obtaining the features for each activity segment in the dataset, we normalize the feature vectors by mean and variance of each feature. That is, the mean of each feature is subtracted from it and divided by its variance to obtain the normalized feature set. We include this step in our framework to ensure that each feature has a zero mean and unit variance.

### 3.5. Comparisons with Existing HAR Datasets

This section presents a comparative analysis of our dataset with other publicly available HAR datasets. We choose the datasets that focus on the activities that are common to our dataset and use either smartphones or wearables as their data collection device. Table 5 summarizes the major characteristics of the datasets. All the previous datasets use a single modality of sensing, i.e., the accelerometer. In contrast, the proposed dataset is the first one to integrate data from accelerometer, gyroscope, and stretch sensors. Having data only from the accelerometer limits previous datasets to fixed-length windows, as shown in the last column of the table. The only exception is the DU-MD dataset [39], which reports variable-length segments. However, variable-length segments in DU-MD are obtained manually, thus making the approach unsuitable for runtime algorithms. The proposed dataset overcomes this problem by using the stretch sensor data to enable variable-length segments that can be obtained at runtime. We acknowledge that the number of user subjects in our dataset is lower compared to some previous studies. We plan to resolve this by continuing to augment our dataset in the future.

## 4. Classifier Design

### 4.1. Supervised Learning Algorithms for HAR

The offline phase of our framework assigns a label to each activity window in the feature set. Then, we use a supervised learning framework that uses the labeled data to train a classifier. The trained classifier is used for inference at runtime. Since online learning for new users is one of our most important goals, we use a neural network (NN) to perform activity classification. We evaluate the accuracy of the NN architecture using w-HAR and other commonly used HAR datasets. We also compare the accuracy of the NN with the most commonly used HAR classifiers, such as k nearest neighbors (k-NN), support vector machine (SVM), decision tree, and random forests.

#### 4.1.1. Design Space Exploration for the Neural Network

Choosing an appropriate structure for the NN is crucial to balance the classification accuracy with the resource requirements of the wearable device. A larger NN achieves a higher accuracy while increasing the memory and processing requirements of the device. Moreover, a larger neural network may also lead to overfitting to the training data, leading to a lower accuracy on new data samples. Therefore, it is crucial to choose the appropriate structure for the NN such that it is robust to new data while keeping the computational complexity low.

**Figure 4:**
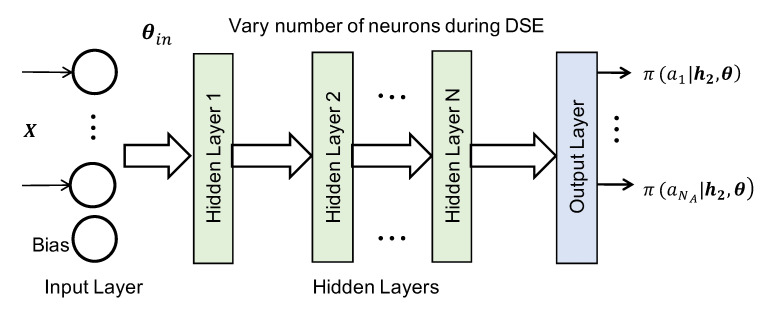
Design space exploration for neural network configuration.

The NN classifier structure is found by performing a design space exploration (DSE) for NNs. The DSE varies the number of hidden layers and the neurons in each hidden layer, as shown in Figure 4. The DSE takes the number of possible layers and number of neurons in each layer as inputs. For example, we can specify that the NN can have one to ten hidden layers. Using these inputs, the DSE trains and tests the accuracy of each possible combination of the number of layers and neurons. The test accuracy is obtained on data not seen during training to ensure that the NN does not overfit. Furthermore, the DSE performs ten iterations of training and testing for each possible combination to ensure robustness. Finally, it automatically chooses the configuration that optimizes the accuracy and resource requirement trade-off as the final classifier. The designer can also analyze the DSE results (accuracy and resource requirements) manually to choose a different combination of layers and the number of neurons.

#### 4.1.2. NN Classifier after DSE

**Figure 5:**
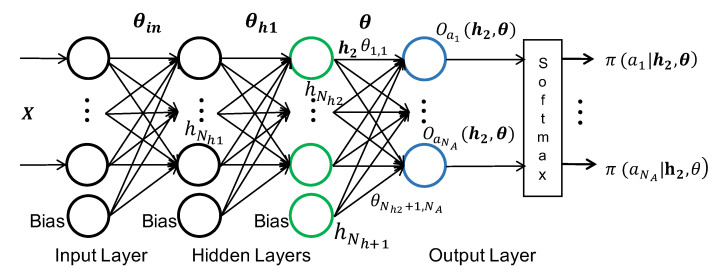
NN architecture used in this paper for activity recognition and online learning.

At the end of the design space exploration, we choose the NN shown in Figure 5 as our classifier. The selected NN has two hidden layers in addition to the input and output layers. The number of neurons in the hidden layers are denoted by $N_{h1}$ and $N_{h2}$, respectively. These parameters are determined after the design space exploration. Both hidden layers use the ReLU activation function and the output layer uses softmax activation. The input feature vector to the NN is denoted by X in Figure 5, and the weights from the input layer to the first hidden layer are denoted by $\theta_{\mathrm{in}}$. Similarly, the weights from the first hidden layer to the second hidden layer are denoted by $\theta_{h1}$. The output layer has one neuron for each of the activities in the w-HAR dataset $a_{i}\in A=\left\{J,L,S,St,W,SU,SD,T\right\},1\leq i\leq N_{A}$, where $N_{A}$ is the number of activities in set A. The weights of the output layer are denoted by θ. The output layer first calculates the activation $O_{a_{i}}\left(X,\theta_{\mathrm{in}},\theta_{h1},\theta \right)$ for each activity $a_{i}$ using the input features X and the NN weights. We express the output activation as a function of the output of the second hidden layer since it helps in the online weight updates described in Section 5. Specifically, the output activation for each activity is:

(2) $$O_{a_{i}}\left(X,\theta_{\mathrm{in}},\theta_{h1},\theta \right)=O_{a_{i}}\left(h_{2},\theta \right)=\sum_{j=1}^{N_{h2}+1}h_{2,j}\theta_{j,i},\;\;\;\;\;\;\;1\leq i\leq N_{A}$$

The term $h_{2,j}$ in Equation (2) is the activation of the jth neuron in the second hidden layer, and $\theta_{j,i}$ is the weight from jth neuron in the second hidden layer to output activity $a_{i}$. Activation $h_{2,j}$ is a function of the input features X, input layer weights $\theta_{\mathrm{in}}$, and the activation of the first hidden layer $h_{1}$. The upper limit of the summation is $N_{h2}+1$ due the fact that the second hidden layer contains $N_{h2}$ neurons and a bias term.

Once we calculate the activation of each output neuron, the probability of each activity $\pi (a_{i}|\;h_{2},\theta )$ is obtained by applying the softmax function to the output activations as:

(3) $$\pi \left(a_{i}|\;h_{2},\theta \right)=\frac{e^{O_{a_{i}}\left(h_{2},\theta \right)}}{\sum_{j=1}^{N_{A}}e^{O_{a_{j}}\left(h_{2},\theta \right)}},\;\;\;\;\;\;\;1\leq i\leq N_{A}$$

In general, the output probabilities $\pi (a_{i}|\;h_{2},\theta )$ can be further expanded to include the input features as one of the arguments, in addition to the NN weights. However, we express them as a function of the second hidden layer output $h_{2}$, since our online learning algorithms focus on updating the weights of the output layer. Given the probability of each activity, the NN chooses the activity with maximum probability as the current activity of the user.

#### 4.1.3. Implementation Cost

We analyze the cost of implementing the w-HAR framework in terms of the number of multiplications and the memory requirements of the NN weights. The majority of multiplications occur in the FFT and NN blocks of w-HAR. Specifically, taking FFT of stretch sensor data results in 264 multiplications while the NN has $121N_{h}+\left(N_{h1}+1\right)\left(N_{h2}+1\right)+\left(N_{h2}+1\right)N_{A}$ multiplications. Finally, the weights of the NN use about 2 kB memory with single-precision floating-point representation. We note that the memory footprint can be reduced by half if we use 16-bit integer weights.

## 5. Online Learning for Human Activity Recognition

We implement the trained NN on the wearable device shown in Figure 1 for online activity recognition. The device is also given to new users whose data is not available during the offline classifier design phase. These new users may have activity patterns that do not match the patterns seen by the NN during the offline training process. Moreover, the activity patterns of the same user may change temporarily due to an injury. Therefore, there is a strong need to develop approaches that continuously update the weights of the NN to adapt to changes in the user or user patterns.

We propose two online learning algorithms for HAR that can be used depending on the level of feedback available from the users since online learning depends critically on feedback from users. The user can provide feedback at varying time scales. For example, when the error is high, the feedback can be given after every activity classification, e.g., every step during walking. As the accuracy of the classifier improves, the frequency of the feedback can be reduced such that the feedback is provided after completing a set of activities, such as walking and sitting. In such cases, the feedback will be provided for multiple activity segments. When the user can indicate whether the inferred activity is correct or wrong, we use the policy gradient algorithm. In contrast, when the user can provide the actual label for the misclassified activities, we incrementally update the weights of the neural network using supervised learning. If the user does not provide any feedback after an activity, the weights of the NN are not updated. In the following, we provide details on each of the above approaches.

### 5.1. Policy Gradient Algorithm for Online Weight Updates

Supervised learning methods are not suitable for online learning if the user is unable to provide the activity labels for misclassifications. Instead, the user only indicates if the classification is correct or wrong. Reinforcement learning is a powerful technique that enables online learning when a correct or wrong feedback is available [19]. Reinforcement learning achieves this by updating the parameters of a policy, such as a neural network, as a function of the feedback received from the user. Common reinforcement learning approaches include Q-learning and policy gradient algorithms [19]. Q-learning algorithm is not suitable for wearable devices since it has to maintain a table of Q-values, which can incur significant memory cost. In contrast, the policy gradient algorithm directly learns the policy, i.e., activity probabilities in Equation (3), without the need to store a Q-table. Therefore, we use the policy gradient algorithm when the feedback only indicates if the classification is correct or wrong. We define the state, action, policy, and the reward for the policy gradient algorithm as follows.

**State:** The state space in the RL framework is given by the stretch sensor and accelerometer data in a segment. The state space is continuous since the sensors can take any real number within their operating limits. The state information from the stretch sensor and accelerometer is then processed by feature generation algorithms to obtain the input feature vector X for the NN.

**Policy:** The activity probabilities $\pi (a_{i}|h_{2},\theta )$ that are generated at the output of NN form the policy in the RL framework. It is obtained by propagating the input features through the NN.

**Action:** We use the activity of the user in a given segment as the action for the policy gradient algorithm. As described in Section 4.1.2, it is obtained by choosing the activity with the maximum probability.

**Reward:** RL techniques give a positive reward when the action is correct, while a negative reward is given for incorrect actions. Following this, we set the reward as +1 when the activity is classified correctly. Otherwise, we set the reward to +1. Moreover, following common RL terminology [19] we define an epoch as the set of segments over which the reward is given, i.e., the number of segments for which the feedback is given. Similarly, each training period is defined as an episode. For example, if a user wears the device for ten minutes before turning it off, the ten minute period forms an episode. Each episode includes a set of epochs for which the reward is given.

**Objective:** The goal in reinforcement learning is to maximize the value function of a state. The value function is given by the sum of rewards obtained starting at the state and taking actions using the policy until the episode ends. Following this, the goal of the policy gradient algorithm is to maximize the total reward J(θ) obtained over an episode. The total reward is a function of the classifier weights since we modify the weights to update the policy towards higher rewards.

**Policy Gradient Weight Updates:** RL techniques often update all the policy weights after every epoch [19]. This approach is beneficial when we start from a completely untrained network with random weight initialization. However, in the w-HAR framework, we start with a NN that is trained offline using the data available at design time. It has been that shown initial layers of a trained network provide general features that are applicable to a wide range of users [42]. Therefore, we update only the weights of the output layer θ in our RL framework. As a result, the NN is able to use the general features learned in the offline phase and minimize the computational cost at runtime.

The policy gradient approach uses the gradient of the objective function with respect to the policy parameters to update the weights. As described above, we use the value function as the objective function. The gradient of the value function has been shown to be proportional to the gradient of the policy in [19]. Using this property, we can write the weight update equation as:

(4) $$\;\theta_{t+1}≐\theta_{t}+\alpha r_{t}\frac{\nabla_{\theta }\pi \left(a_{t}|h_{2},\theta_{t}\right)}{\pi (a_{t}|\;h_{2},\theta_{t})},\;\;\alpha :\mathrm{Learning}\;\mathrm{rate}$$

where $\theta_{t}$ and $\theta_{t+1}$ are the current and updated weight matrices, respectively. Similarly, $a_{t}$ is the activity classified by the classifier at time *t*, $r_{t}$ is the current reward for the policy, and $h_{2}$ is the vector of outputs generated at the second hidden layer. In order to obtain the new weights using the update rule, we need to calculate the gradient of the policy with respect to the output layer weights θ. The value of this gradient depends on whether a given weight connects to the neuron that corresponds to the output activity $a_{t}$ or not. To this end, we first generate two sets $S_{t}$ and $\bar{S_{t}}$. The set $S_{t}$ includes the weights that directly connect to the neuron corresponding to the output activity $a_{t}$, while $\bar{S_{t}}$ contains the other weights. Note that each weight belongs to only one of the sets, i.e., they are disjoint. Using the definition of $S_{t}$ and $\bar{S_{t}}$, the weight update rule can be written as:

(5) $$\theta_{t+1,j,i}≐\left\{\begin{array}{cc} \theta_{t,j,i}+\alpha r_{t}\left(1-\pi \left(a_{t}|\;h_{2},\theta_{t}\right)\right)\cdot h_{2,j} & \theta_{t,j,i}\in S_{t}\\ \theta_{t,j,i}-\alpha r_{t}\pi \left(a_{i}|\;h_{2},\theta_{t}\right))\cdot h_{2,j} & \theta_{t,j,i}\in \bar{S_{t}} \end{array}\right.$$

The detailed proof of the update rule can be found in [6].

### 5.2. Online Updates with Incremental Supervised Learning

Online learning using the policy gradient algorithm is most useful when the activity labels are not available. While it can be used when activity labels are available, the convergence rates of the policy gradient algorithm are lower when compared to supervised learning. Therefore, we use supervised learning for performing incremental weight updates when the user can provide activity labels at runtime, as outlined in Algorithm 1. The algorithm takes the offline trained weights of the NN as its input. The first step in the algorithm is to initialize a buffer *B* of size *M* that is used to store the training data for weight updates. With this initialization, we start the online phase of the algorithm. For each activity segment *t*, we first obtain the feature vector $X_{t}$ and use it with the weights to determine the activity probabilities (lines 4–5). Then, we assign the activity with the maximum probability as the output activity. This activity is shown to the user who then provides the actual activity label $a_{t}^{*}$ to the algorithm. If the actual activity label $a_{t}^{*}$ does not match the activity output of the NN, we store both the feature vector $X_{t}$ and the label $a_{t}^{*}$ in the buffer *B* (lines 7–10). Otherwise, we proceed to the next activity. This process continues until the buffer is full. Once the buffer is full, we use the training data in the buffer to update the weights of the NN using the backpropagation algorithm. Similar to the policy gradient approach, we update only the weights in the output layer. Finally, we reset the data in buffer *B* so that training data from the updated network can be collected.
**Algorithm 1:** Weight Update via Supervised Learning
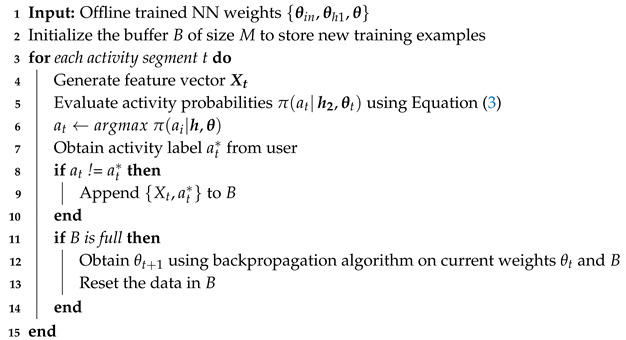


In summary, the weights of the output layer are updated online using either of the two algorithms after user feedback. We note that the weights of the hidden layers can be updated similarly by computing the gradient of the hidden layers with respect to their weights. Detailed results for the improvement in accuracy using the online learning approaches and a comparison among them are presented in Section 6.5.

## 6. Experimental Evaluation and Discussions

### 6.1. Experimental Setup

We implement the proposed HAR framework on the TI-CC2650 [43] IoT device. We place the TI-CC2650 device on the ankle while the flexible stretch sensor is worn on the knee. The stretch sensor transmits the data to the TI-CC2650 [43] IoT device that processes the data to perform the activity recognition. The recognized is then transmitted to a host device, such as a smartphone. We transmit only the activity classification since transmission of the raw data incurs a higher communication overhead.

**Training, cross-validation, and test data split:** We first divide the users into two sets, *offline training*, and *online training*. The offline training set includes 18 users, while the online training set includes the remaining 4 users. The users in the offline training set are used to train the neural network classifier. Within this dataset, we reserve 60% data for training, 20% data for cross-validation, and 20% data for test. The trained classifier is then used to perform activity classification for the online training users and update the weights of the network. Furthermore, to ensure the robustness of the proposed framework, we create a total of 30 combinations of *offline training*, and *online training* sets. In each combination, we ensure that number of common users is minimal.

### 6.2. Neural Network Design Space Exploration

**Figure 6:**
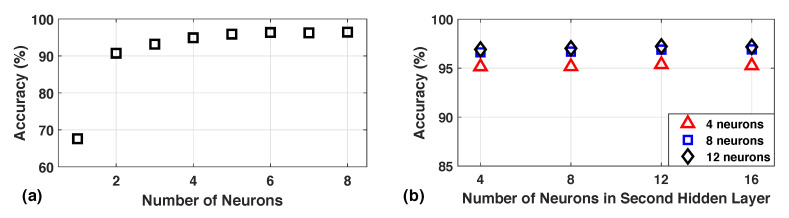
Accuracy of (**a**) one hidden layer, and (**b**) two hidden layer networks with varying number of neurons in the hidden layers.

The trained NN classifier is used to identify the activities of users at runtime. It is also updated at runtime using the online learning algorithms in Section 5. To achieve online classification and training, the NN must be implemented on the wearable devices that have limited memory resources. Therefore, the NN should minimize the number of weights without sacrificing the classification accuracy. In order to choose the neural network structure for HAR, we perform a design space exploration with a single hidden layer and two hidden layer networks. In each of the networks, we vary the number of neurons in the hidden layers to study the effect on accuracy and memory requirements. Figure 6a shows the change in accuracy as we increase the number of neurons in the hidden layer for a NN with a single hidden layer. We see that the accuracy of the network saturates at around 93% after 4 neurons in the hidden layer. The memory requirement *M* for this network is given by $M=121*N_{h}+N_{h}*8$, where $N_{h}$ is the number of neurons in the hidden layer. The equation shows that the addition of a single neuron in the hidden layer leads to 120 additional weights. Therefore, addition of neurons to the NN incurs a high memory cost while providing marginal improvements in accuracy.

Next, we analyze the accuracy obtained by NNs with two hidden layers. We first fix the number of neurons in the first layer and then vary the number of neurons in the second layer. We repeat this for different number of neurons in the first layer. Figure 6 shows the accuracy of the NN when we fix the first layer neurons to 4, 8, and 12, respectively. The three types of markers represent the neurons in the first layer while the x-axis shows the number of neurons in the second layer when the first layer is fixed. All the networks achieve similar accuracy with 4-neuron networks having a slightly lower accuracy. At the same time, the 4-neuron networks come with a significant memory advantage. When we compare the networks with one and two hidden layers, the two-layer networks achieve better accuracy with a slightly higher memory cost. This is because the memory cost additional of neurons in the second layer is much lower than in the first layer. Therefore, in our implementation, we choose a NN with 4 neurons in the first layer and 8 neurons in the second layer. We choose this over a network with just 4 neurons in the second layer as it provides a more robust operation. The chosen network provides a 95.32% accuracy with a 2 kB memory requirement.

### 6.3. Accuracy Analysis of the Neural Network

#### 6.3.1. Confusion Matrix

Once we finalize the structure of the NN classifier, we move on to train the NN using the data from 18 users reserved for offline training. We analyze the accuracy of the offline training using a confusion matrix, as shown in Table 6. The confusion matrix shows the accuracy for all the seven activities and transitions. The rows correspond to the true activity label, while the columns show the classification output. Consequently, the diagonal of the confusion matrix gives the accuracy for each activity. For example, the third column of the third row shows that the accuracy for classifying the sit activity is 96.4%. Similarly, the fourth column of the third row shows that 2.98% of sit activity windows are misclassified as stand. In addition to the accuracy, the confusion matrix also shows the number of segments of each activity next to the activity name on each row.

From the confusion matrix, we see that the NN achieves accuracy greater than 94% for all activities except transition. The accuracy is lower for transitions as each transition window includes features from two distinct activities. This is tolerable since transitions can be implied from activity labels before and after a transition. Finally, we note that all 30 combinations of training user sets achieve similar confusion matrices. Therefore, we do not report the confusion matrix for each combination.

#### 6.3.2. Comparison with Other Classifiers

One to one comparison with existing approaches for HAR is not feasible since they use devices, datasets, and activities that are different from our study. Therefore, we implement commonly used supervised learning classifiers on our dataset and compare the accuracies. Table 7 shows a comparison of the accuracy of commonly used classifiers. We see that the proposed neural network classifier achieves a competitive accuracy when compared to the other classifiers. While the other classifiers achieve a slightly higher accuracy then the NN, they are not amenable to online learning. In contrast, the proposed NN can be efficiently updated at runtime to enable online learning for HAR, which is one of the focus areas of this work.

#### 6.3.3. Robustness of the NN Classifier

**Figure 7:**
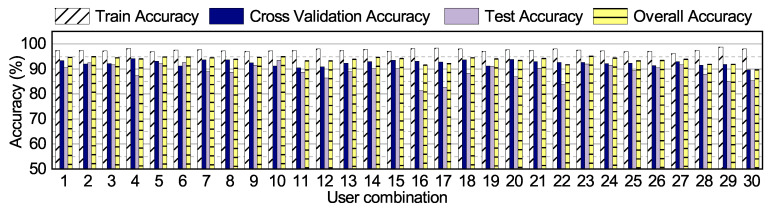
Comparison of recognition accuracy with different combinations of users for training.

The proposed NN classifier must be robust to input from different users. Therefore, we perform an accuracy analysis with the 30 user combinations described in Section 6.1. We first train with 60% of the data from the 18 users present in the *offline training* set of each user combination. The training phases also uses 20% of the data in each set for cross-validation during training. After training a NN with each user combination, we test them with the remaining 20% data and 4 unseen users (*online training* set). The accuracy results for the 30 classifiers obtained in this analysis are summarized in Figure 7. We see that all the user combinations achieve training accuracy higher than 95% and cross-validation accuracy greater than 90%. Moreover, most of the combinations achieve test accuracy higher than 90%. A few of the user combinations, such as 16, 17, and 30, have lower test accuracy. This can be attributed to the fact that the activity signatures observed in the training set of 18 users do not capture the activity signatures of the 4 test users. We can overcome this issue by either including the users in the training set or applying online learning, as described in the Section 6.5.

### 6.4. Evaluation of the Neural Network Architecture with Other Datasets

This section evaluates the accuracy of the proposed NN architecture design on four publicly available HAR datasets, as summarized in Table 8. Each row of the table contains the neural network structure and accuracy for the corresponding dataset. The accuracy is obtained by performing 5-fold cross-validation of each dataset. In the following, we provide a brief description of the dataset and classification accuracy on each dataset.

**WISDM** [11]: The WISDM dataset is one of the most commonly used datasets for HAR research. It contains smartphone accelerometer data for six activities collected from 29 users. The sampling frequency of the accelerometer is 20 Hz. The authors of the dataset recommend using 10 s windows to generate the features. Following this, we first generate DWT and variance features of the accelerometer data. Since stretch sensor data is not available for the WISDM dataset, we also generate FFT, minimum, and maximum value for accelerometer data. As shown in Table 8, the proposed NN architecture achieves 95% accuracy for the WISDM dataset using these features.

**UCI HAR** [16]: UCI-HAR is another commonly used dataset in the HAR literature. It also contains smartphone accelerometer data for six activities collected from 30 users. The sampling frequency used in this dataset is 50 Hz. The UCI-HAR dataset provides pre-processed data with time and frequency domain features for each activity window. When trained with these features, our NN architecture provides 98% accuracy.

**Shoaib et al.** [40]: The dataset from Shoaib et al. includes smartphone IMU data for ten subjects performing seven activities. In contrast to other datasets, Shoaib et al. collect data at five body positions (right pocket, left pocket, on the belt, right upper arm, and right wrist). Of these, we choose the data from the left pocket for the accuracy analysis because it is close to our experimental setup. Moreover, we choose the left pocket over the right pocket to show the NN classifier’s applicability to data from the left leg as well. After choosing the location, we obtain DWT, FFT, and statistical features for the accelerometer using the procedure described for the WISDM dataset. With the generated features, our NN architecture achieves 95% accuracy.

**UniMib SHAR** [15]: This dataset contains accelerometer readings from 30 users and nine physical activities. The authors of the dataset provide pre-processed and segmented data for the activities. Using these segments, we generate the DWT, FFT, and statistical features for the accelerometer data. Unlike the other three datasets, the NN architecture obtained in Section 4.1 has lower accuracy. Therefore, we perform a DSE of NN architectures for the UniMib SHAR dataset. We note that the DSE step takes less than one hour to complete. At the end of the DSE, we choose a NN consisting of one hidden layer with 12 neurons. The chosen NN architecture achieves an accuracy of 90% for the UniMib SHAR dataset. The accuracy is slightly lower than the other datasets because some of the activities have very similar patterns, making it harder to classify them.

In summary, the proposed NN architecture, along with the DSE step, provides high classification accuracy on commonly used HAR datasets. This shows that the proposed approach is robust to a wide range of activities and datasets.

### 6.5. Online Learning with New Users

**Figure 8:**
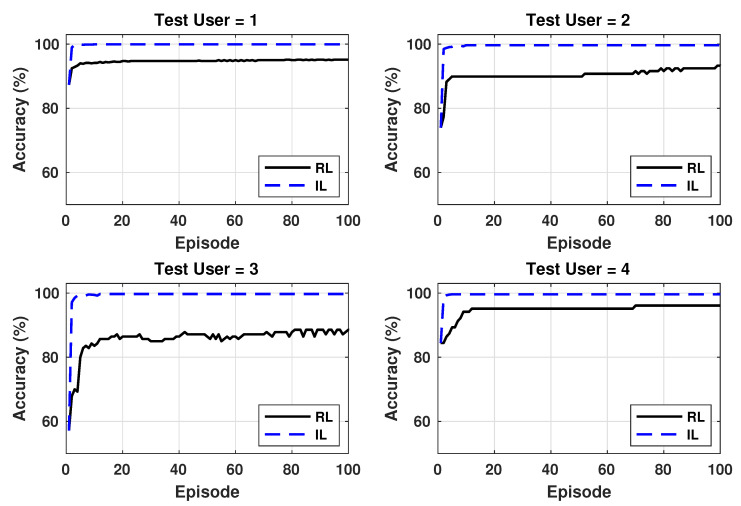
Comparison of reinforcement learning and incremental learning.

We use the NN classifiers trained for each of the 30 user combinations to recognize the activities of the users not included in the training set. As we see in the previous section, the test accuracies for some of the user combinations are lower than 90%. Therefore, we use the proposed online learning algorithms to adapt the weights of the classifiers to the new users. Figure 8 shows the improvement in accuracy obtained by the reinforcement learning and incremental supervised learning for four users not see during training. The initial NN for these users is obtained from user combination 25 that has greater than 95% training accuracy. However, we see that the initial accuracy for all four users is lower than the accuracy for training users. In particular, the accuracy for user 10 is only about 60%. Starting with this initial accuracy, we apply the online learning algorithms. The episodes on the x-axis correspond to an iteration of weight updates using either the reinforcement learning or incremental learning approaches. We run the online updates for a total of 100 episodes in Figure 8. We note that the data set of a particular user is reused in each episode of online learning. As more data becomes available for each user, we can use different subsets of data in each episode. We see that the online learning algorithms provide a consistent improvement in accuracy for all the new users. For instance, the policy gradient algorithm improves the accuracy of user 19 from about 83% to 95%, while the incremental supervised learning approach improves the accuracy to almost 100%. Similar improvements are observed for other users as well. We also see that the incremental supervised learning approach improves the accuracy much faster and to a higher level when compared to the policy gradient approach. This is expected since it uses the actual activity labels instead of an indirect reward. Therefore, it is more beneficial to use the incremental supervised learning algorithm when the user can provide the actual activity labels. We note that the user feedback can be obtained periodically from the user by prompting them to enter the information on a smartphone app. The frequency of the feedback can be reduced as the classifier adapts to the user.

Table 9 provides a summary of the improvement obtained through online learning for all 30 user combinations. The table contains a set of three columns for each user in the *online training* set. Within each set of columns, we show the initial accuracy at the beginning of online training, the final accuracy obtained with reinforcement learning, and the final accuracy obtained with incremental learning. We see that the incremental learning approach achieves accuracy greater than 99% for all user combinations. We can attribute this to the fact that the neural network adapts to the patterns of the user, thus providing personalized HAR. Reinforcement learning also achieves accuracy greater than 90% for most of the user combinations and test users. For a few of the users, such as test user 1 in combination 16, the accuracy improvement with reinforcement learning saturates. This limitation can either be resolved using incremental learning or updating all the layers in the network. In summary, the online learning algorithms proposed in this paper consistently improve the accuracy for new users and provide a path to personalized HAR.

## 7. Conclusions

Human activity recognition has wide-ranging applications from movement disorders to patient rehabilitation to activity promotion in the general population. Successful research in HAR critically depends on the availability of open-source datasets. In order to address this need, we presented a w-HAR, an open-source dataset for human activity recognition that, *for the first time*, includes data from both wearable stretch and IMU (accelerometer + gyroscope) sensors. We provide three versions of the sensor data as part of w-HAR. The first version provides raw sensor data that allows researchers to develop their own algorithms for all steps of HAR. Secondly, we provide segmented data that gives researchers the freedom to just focus on feature generation and classification. Finally, we also provide a baseline feature set for researchers who want to focus only on classifier development. The baseline feature set includes the discrete wavelet transform of the accelerometer and fast Fourier transform of the stretch sensor data. The baseline features do not include the gyroscope data. However, researchers can use the raw gyroscope data to generate features from it. After presenting the dataset, we performed a DSE of neural network classifiers for HAR. After the DSE, we obtained a NN classifier that achieved 95% accuracy for user data available at design time. Then, we introduced two online learning algorithms to continuously improve the classifier weights for new user subjects as a function of user feedback. The online learning algorithms improved the accuracy for new users by as much as 40%.

The HAR framework presented in this paper can be easily extended to other activities, such as jogging, bending down, falling, and cycling. The addition of new activities will require data collection for a subset of users and training the classifier. We can also perform DSE to identify new NN architectures if the current architecture cannot achieve high accuracy. Once the classifier is trained with a subset of users, it will adapt to new users with the help of online learning algorithms proposed in this paper. As part of our future work, we plan to grow the dataset with additional subjects and activities. Specifically, we plan to include users with limited mobility in the dataset to provide a wider range of coverage. We also plan to develop online learning algorithms that can adapt efficiently to movement disorder patients.

## Figures and Tables

**Table 1:** Summary of HAR classifiers.

Ref.	Device	Sensors	No. of Activities	Classifier	Acc. (%)	Power	Online Learning
[20]	Smartphone	Accelerometer	6	KNN	95	-	No
[26]	Wearable	3 Accelerometers	12	KNN, RF, SVM	99, 99, 95	2.7 W	No
[29]	Wearable, smartphone	Heart rate, resp. rate, accelerometer	4	Decision Tree	96	-	No
[30]	Wearable	Accelerometer	7	Decision tree, ExtraTrees	92	9.5 mW	No
[28]	Smartphone	Accelerometer	6	CNN	90–97	-	No
[31]	Wearable	Piezoelectric harvester	5	KNN	85	3.2 mW	No
w-HAR	Wearable	Accelerometer, stretch sensor	7	Neural Network	95%	12.5 mW	Yes

**Table 2:** Activities performed by users in the w-HAR framework.

• Jump (J)	• Lie Down (L)	• Sit (S)
• Stand (St)	• Walk (W)	• Stairs Up (SU)
• Stairs Down (SD)	• Transition (T) between the activities	

**Table 3:** Experimental protocol for the HAR dataset.

Experiment 1	Experiment 2	Experiment 3	Experiment 4	Experiment 5	Experiment 6	Experiment 7
Stand 30 s Jump 3 times Stand 30 s	Stand 10 s Sit 30 s Stand 10 s Jump 3 times Sit 30 s	Stand 10 s Walk 40 steps Stand 10 s	Stand 10 s Jump 3 times Walk 40 steps Sit 20 s	Stand 10 s Sit 10 s Lie down 30 s Sit 10 s	Stand 10 s Walk down stairs Stand 10 s	Stand 10 s Walk up stairs Stand 10 s

**Table 4:** Summary of the number of segments in each activity.

Activity	Segments	Activity	Segments
Jump	458	Walk	2007
Lie down	474	Stairs up	109
Sit	696	Stairs down	99
Stand	620	Transition	277

**Table 5:** Comparison with existing HAR datasets.

Dataset	Device	Sensors	No. of Subjects	No. of Activities	Variable Length Segments
DU-MD [39]	Wearable	Accelerometer	33	7	Yes, manually
Shoaib et al. [40]	Smartphone	IMU	10	7	No
UCI HAR [16]	Smartphone	Accelerometer	30	6	No
Ugulino et al. [41]	Wearable	Accelerometer	5	4	No
UniMiB SHAR [15]	Smartphone	Accelerometer	30	9	No
USC-HAD [18]	Wearable	Accelerometer	14	12	No
WISDM [11]	Smartphone	Accelerometer	29	6	No
w-HAR	Wearable	IMU, Stretch sensor	22	7	Yes

**Table 6:** Confusion matrix for 18 training users.

	Jump	Lie Down	Sit	Stand	Walk	Stairs Up	Stairs Down	Transition
Jump (392)	94.6%	0.00	0.26%	0.00	4.08%	0.00	0.26%	0.77%
Lie Down (466)	0.00	99.8%	0.21%	0.00	0.00	0.00	0.00	0.00
Sit (637)	0.00	0.00	96.4%	2.98%	0.00	0.00	0.00	0.63%
Stand (566)	0.00	0.00	2.47%	95.1%	0.00	0.00	0.00	2.47%
Walk (1598)	0.75%	0.00	0.25%	1.23%	94.5%	0.19%	0.69%	2.38%
Stairs Up (82)	0.00	0.00	0.00	0.00	0.00	96.3%	3.66%	0.00
Stairs Down (79)	0.00	0.00	0.00	0.00	3.80%	0.00	96.2%	0.00
Transition (243)	1.23%	0.00	2.47%	2.88%	2.88%	0.00	0.00	90.5%

**Table 7:** Comparison of accuracy for different classifiers.

Classifier	Train Acc. (%)	Test Acc. (%)	Overall Acc. (%)
Random Forest	100.00	95.60	99.12
C4.5	98.67	91.70	97.28
k-NN	96.76	94.39	96.29
SVM	99.01	93.53	97.91
Our NN	96.24	91.71	95.32

**Table 8:** Evaluation of the proposed neural network architecture with commonly used datasets.

Dataset	No. of Hidden Layers	No. of Neurons in Hidden Layers	No. of Activities	Accuracy (%)
WISDM [11]	2	4, 8	6	94
UCI HAR [16]	2	4, 8	6	98
Shoaib et al. [40]	2	4, 8	7	95
UniMiB SHAR [15]	1	12	9	90

**Table 9:** Comparison of accuracy improvement with online learning.

User Comb.	Test User 1 Accuracy (%)			Test User 2 Accuracy (%)			Test User 3 Accuracy (%)			Test User 4 Accuracy (%)		
	Init.	RL	IL	Init.	RL	IL	Init.	RL	IL	Init.	RL	IL
1	93.7	97.5	99.7	93.2	98.9	99.9	88.2	95.1	99.7	75.0	82.1	99.7
2	91.1	92.4	99.6	83.3	86.1	99.2	82.4	94.1	98.2	91.1	95.8	99.8
3	93.7	97.5	99.5	92.2	98.4	99.9	76.1	87.7	99.9	88.4	95.3	99.9
4	91.1	100.0	99.9	86.9	94.6	100.0	58.8	85.3	99.6	80.6	94.2	99.6
5	97.5	97.5	99.7	96.2	97.5	99.9	82.5	96.0	99.8	84.4	91.1	99.1
6	86.4	92.0	99.7	97.2	97.2	99.7	97.1	98.8	100.0	86.3	94.3	99.9
7	94.3	98.9	99.5	88.2	94.1	98.8	91.4	94.5	99.8	60.8	89.2	99.8
8	92.0	93.2	99.8	92.2	95.3	99.8	79.3	95.3	99.9	87.4	96.1	99.7
9	89.6	94.4	99.7	94.4	97.2	99.4	93.3	96.9	99.8	91.3	97.1	99.8
10	93.1	97.9	99.8	88.2	94.1	98.8	98.4	99.2	99.8	96.8	98.7	100.0
11	66.0	93.8	99.9	80.2	93.2	99.8	90.1	95.9	99.9	87.3	96.8	99.7
12	84.7	92.4	99.8	79.7	94.4	100.0	54.9	65.7	99.6	82.2	88.9	99.1
13	69.3	84.3	99.7	88.9	91.7	98.9	80.2	96.1	99.9	93.6	99.4	99.9
14	56.4	82.9	99.7	76.5	94.1	97.6	89.2	94.9	99.9	84.4	91.1	99.3
15	81.4	87.1	99.8	93.2	96.4	99.8	98.4	99.6	99.8	67.6	91.2	99.6
16	48.4	66.8	99.9	55.7	81.4	99.9	92.6	95.1	99.8	88.9	95.2	99.8
17	43.0	67.7	99.9	96.3	98.4	99.9	86.4	94.2	99.7	88.9	91.1	99.1
18	92.4	94.1	99.7	86.1	91.7	98.9	52.9	72.5	99.9	91.3	91.3	99.7
19	89.1	92.4	99.7	87.0	93.8	99.9	85.9	91.4	99.8	96.2	99.4	99.9
20	95.0	95.8	99.7	64.1	80.1	99.9	82.4	88.2	98.2	87.9	94.8	99.8
21	88.6	98.1	99.9	88.2	94.1	98.8	81.6	94.2	99.6	85.7	92.1	99.8
22	94.4	99.3	100.0	42.7	59.6	99.9	89.8	96.6	99.7	96.8	99.4	99.9
23	89.1	97.8	99.8	92.4	95.0	99.7	92.0	94.3	99.7	82.2	91.1	99.3
24	78.3	100.0	100.0	90.1	99.0	99.9	92.0	94.5	99.8	90.4	94.9	99.9
25	87.3	95.2	100.0	73.9	92.4	99.7	57.1	87.1	99.7	84.5	96.1	99.6
26	91.9	95.4	99.9	85.5	94.7	99.9	88.9	97.2	98.9	88.9	97.8	99.6
27	92.0	96.4	100.0	89.1	100.0	100.0	99.2	99.2	99.9	92.9	96.0	99.7
28	83.7	88.5	100.0	86.7	98.8	99.9	87.4	94.1	99.7	95.9	97.9	99.9
29	68.3	85.9	100.0	76.1	100.0	99.8	55.9	73.5	99.7	63.1	93.0	99.9
30	81.1	90.3	100.0	72.2	95.0	99.9	54.3	94.4	99.8	66.9	95.5	99.9

## Abbreviations

The following abbreviations are used in this manuscript:

HAR	Human activity recognition
FFT	Fast Fourier transform
DWT	Discrete wavelet transform
RL	Reinforcement learning
NN	Neural network
DSE	Design space exploration

## Appendix A. Pseudo-Code for the Segmentation Algorithm

The segmentation algorithm takes the streaming stretch data as its input. Using this input, it first calculates the five-point derivative to track the trend of the sensor value as:

(A1) $$s^{\prime }\left(t\right)=\frac{s(t-2)-8s(t-1)+8s(t+1)-s(t+2)}{12}$$

where s(t) and $s^{\prime }\left(t\right)$ are the stretch sensor value and its derivative time step *t*, respectively. Using the derivative, we know that the trend as increasing when the derivative is positive, decreasing when the derivative is negative, and flat otherwise. We can just use this value of trend to mark segments; however, looking at a single point can lead to false alarms. Therefore, the algorithm looks at a short window of the derivative to determine the trend. In our specific implementation, we change the trend of the data only if three consecutive derivative values indicate a new trend (lines 10–18 in Algorithm A1). For example, if the current trend is increasing, we require that at least three derivative values be zero before setting the new trend as flat. This helps in filtering out glitches in the data. Using this, we make a new segment whenever the trend changes from flat or decreasing to increasing. The final consideration in the segmentation algorithm is to bound the length of the windows from above and below. In order to bound the length of a segment from below, we start looking for new segments only after a fixed amount of time (one second in our implementation) has passed since the last window. The minimum window length check is performed by the if statement in line 19 of Algorithm A1. Lower bounding the window size saves computation time and power by preventing windows that are shorter than normal activity lengths. Similarly, we enforce a maximum window length to improve robustness in case a local minimum is missed. We use $t_{max}=3$ s as the upper bound since it is long enough to cover all transitions.

**Algorithm A1:** Segmentation Algorithm
